# Loss of Clustered Protocadherin Diversity Alters the Spatial Distribution of Cortical Interneurons in Mice

**DOI:** 10.1093/texcom/tgaa089

**Published:** 2020-11-25

**Authors:** Nicholas Gallerani, Edmund Au

**Affiliations:** 1 Department of Pathology & Cell Biology, Columbia University Irving Medical Center, New York, NY 10032, USA; 2 Department of Rehabilitative Medicine and Regeneration, Columbia University Irving Medical Center, New York, NY 10032, USA; 3 Columbia University Irving Medical Center, New York NY, 10032, USA

**Keywords:** clustered protocadherins, cortical interneurons, spatial distribution

## Abstract

Cortical interneurons (cINs) are locally projecting inhibitory neurons that are distributed throughout the cortex. Due to their relatively limited range of influence, their arrangement in the cortex is critical to their function. cINs achieve this arrangement through a process of tangential and radial migration and apoptosis during development. In this study, we investigated the role of clustered protocadherins (cPcdhs) in establishing the spatial patterning of cINs through the use of genetic cPcdh knockout mice. cPcdhs are expressed in cINs and are known to play key functions in cell spacing and cell survival, but their role in cINs is poorly understood. Using spatial statistical analysis, we found that the 2 main subclasses of cINs, parvalbumin-expressing and somatostatin-expressing (SST) cINs, are nonrandomly spaced within subclass but randomly with respect to each other. We also found that the relative laminar distribution of each subclass was distinctly altered in whole α- or β-cluster mutants. Examination of perinatal time points revealed that the mutant phenotypes emerged relatively late, suggesting that cPcdhs may be acting during cIN morphological elaboration and synaptogenesis. We then analyzed an isoform-specific knockout for pcdh-αc2 and found that it recapitulated the α-cluster knockout but only in SST cells, suggesting that subtype-specific expression of cPcdh isoforms may help govern subtype-specific spatial distribution.

## Introduction

The correct spatial distribution of neurons can be critical to brain function. This is true in the case of cortical interneurons (cINs) of the neocortex, which are locally projecting inhibitory cells that carry out key processes, including controlling cortical rhythmicity (Sohal et al. 2009), boosting signal salience (Lee et al. 2012; Song et al. 2020), and regulating spike timing (Tiesinga et al. 2008). Diverse subclasses of cINs are found throughout the neocortex, where they establish repetitive microcircuit motifs (Tremblay et al. 2016).

How interneuron subclasses appropriately distribute and establish an inhibitory network poses a difficult logistical problem. Developmentally, cINs arise from distal progenitor domains and migrate long distances into the cortex (Wamsley and Fishell 2017). Following migration, cIN number is reduced through apoptotic cell death (Southwell et al. 2012) and cINs extend complex, branched dendritic and axonal arbors that overlap considerably with neighboring cINs, enabling a single cIN to inhibit multiple pyramidal cells (PC), and for a single PC to be innervated by multiple cINs (Fino and Yuste 2011; Packer et al. 2013). How these stages are orchestrated to allow cINs to establish a distributed network of inhibition is poorly understood.

An appealing molecular candidate that could mediate this process is a family of adhesion molecules known as clustered protocadherins (cPcdhs). cPcdhs are arranged in 3 gene clusters that encode 58 distinct isoforms of α-, β-, and γ-cPcdhs (Kohmura et al. 1998; Wu and Maniatis 1999; Chen and Maniatis 2013). They impart neurons with single-cell identity by differential (and largely stochastic) expression of cPcdhs, which form combinatorial cPcdh recognition complexes (Esumi et al. 2005; Hirano et al. 2012; Brasch et al. 2019). These molecules mediate crucial circuit formation functions (Mountoufaris et al. 2017) including dendritic self-avoidance (Lefebvre et al. 2012), axonal tiling (Chen et al. 2017), and cell survival (Wang et al. 2002; Hasegawa et al. 2016; Garrett et al. 2019). Deficits in α-cPcdh diversity can cause detectable changes in sensory processing (Yamagishi et al. 2018). cPcdhs are expressed in cINs (Hirano et al*.* 2012; Mancia Leon et al. 2020), and recently, it was demonstrated that γ-cPcdhs regulate programmed cell death in cINs (Mancia Leon et al*.* 2020). However, the role of α- and β-cPcdhs in cINs is still poorly understood. We therefore sought to determine if α- and β-cPcdhs are important for other aspects of cIN development, such as facilitating proper subclasses spatial distribution.

To test this, we developed a method of registering cIN cell body positions in *xy* coordinate space. This allowed us to analyze spatial distribution of the 2 main interneuron subclasses, parvalbumin+ (PV) and somatostatin+ (SST) cINs. Together, they account for ~ 70% of all cINs (Tremblay et al*.* 2016) and share a developmental origin in the medial ganglionic eminence (MGE) (Wamsley and Fishell 2017). The density of cIN processes makes them difficult to directly assess at the population level in vivo, but we reasoned that since interneurons possess locally projecting processes, their cell body placement was a reasonable and accessible proxy for the area of influence of the neuron. The position of the cell body has been similarly used to study neuron spacing in locally projecting inhibitory retinal amacrine cells (Fuerst et al. 2008).

Using this method, we compared cINs in wild-type (WT) mice, whole α-cPcdh knockout (α-KO), and whole β-cPcdh knockout (β-KO) mutant mice and found that loss of cPcdh diversity differentially affects PV and SST cINs. In the adult primary visual cortex (V1) of the α-KO, we found that PV cell density was reduced, whereas SST density was not. Additionally, we observed subclass-specific alterations in laminar distribution of PV and SST populations. Both α- and β-KOs exhibited defects, but the phenotype was most pronounced in V1 of the α-KO. Focusing on V1 in WT and α-KO, we performed an analysis at perinatal time points and found that the mutant phenotype arises after migration and during the late phase of canonical cell death. To further understand how the α-KO differentially affects cIN, we examined pcdh-αc2 (αc2), an α-cPcdh isoform that is nonstochastically expressed (Chen and Maniatis 2013). In the αc2-specific mutant (αc2-KO), the SST phenotype was similar to the α-KO phenotype, but PV cells were unaffected. Taken together, our work suggests that cPcdhs play an important role in the laminar distribution of cINs during perinatal development. Further, our work suggests that subclasses of cINs employ specific cPcdh isoforms (αc2 in the case of SST cells) as they establish a distributed inhibitory network in the cortex.

## Materials and Methods

### Animals

All animal experiments were performed in compliance with established protocols approved by Institutional Animal and Use Committee (IACUC). α-KO and β-KO mice were gifts from Dr Tom Maniatis and are the same strains described previously (Mountoufaris et al*.* 2017). These mice share the same mixed 129 and C57BL/6J genetic background. We used mixed 129 and C57BL/6J mice as a WT control group for comparison to both α-KO and β-KO. The αc2-KO was also a gift from Dr Tom Maniatis and was generated according to methods described in a previous study (Chen et al*.* 2017), but only ablates the Pcdh-αc2 variable exon, rather than both Pcdh-αc1 and Pcdh-αc2. These mice are also mixed 129 and C57BL/6J, but here we used Pcdh-αc2^WT^ littermates as controls. For perinatal experiments, α-KO mice were crossed with BAC-Nkx2.1-Cre (Jackson 008661 C57Bl/6J-Tg(Nkx2-1-cre)2Sand/J); Ai9 (Jackson 007909 B6.Cg-Gt(ROSA)26Sor^tm9(CAG-tdTomato)Hze^/J) mice. Mice heterozygous for these alleles were crossed to generate Nkx2.1-Cre; Ai9; α-KO/α-KO; or α-WT/α-WT mice used in perinatal experiments. We analyzed the following number of mice: (WT for whole cluster: *n* = 7, α-KO: *n* = 10, β-KO: *n* = 8, WT for αc2-KO: *n* = 5, αc2-KO: *n* = 5, postnatal day 3 [P3] Nkx2.1Cre; Ai9; α-WT: *n* = 4, P3 Nkx2.1Cre; Ai9; α-KO: *n* = 5, postnatal day 7 [P7] Nkx2.1Cre; Ai9; α-WT: *n* = 6, P7 Nkx2.1Cre; Ai9; α-KO: *n* = 6, postnatal day 13 [P13] Nkx2.1Cre; Ai9; α-WT: *n* = 5, Nkx2.1Cre; Ai9; α-KO: *n* = 5).

### Histology

Mice were anesthetized with ketamine (87.5 mg/kg) xylazine (12.5 mg/kg) via intraperitoneal injection and transcardially perfused with phosphate-buffered saline (PBS) followed by 4% paraformaldehyde (PFA) in PBS. Brains were postfixed in 4% PFA/PBS overnight at 4°C, embedded in low-melt agarose and then parasagitally sectioned on a Leica VT1000S vibratome (50 μm thickness). Sections were stored at −30°C in an antifreeze solution containing polyethylene glycol, glycerol, and PBS.

### Immunohistochemistry

Sections were transferred from antifreeze solution to PBS and washed 3× in PBS. Heat-induced epitope retrieval was then performed by incubating sections in 10 mM sodium citrate (pH 8.5) at 80°C for 30 min. Sections were then incubated at room temperature in a blocking/permeabilization buffer consisting of 2% w/v nonfat-dried milk, 0.3% Triton X, and 0.01% sodium azide in PBS. Sections were incubated in primary antibodies diluted in 2% normal donkey serum and 0.02% Tween20 in PBS for 24–72 h at 4°C. Primary antibodies used in this study: guinea pig anti-PV (1:750, Immunostar), rat anti-SST (1:300, EMD Millipore), rabbit anti-active caspase 3 (1:1000, Sigma-Aldrich), rabbit anti-serotonin transporter (1:1000, Immunostar). Sections were incubated in secondary antibodies diluted 1:1000 in PBS for 2 h at room temperature. All secondary antibodies were Alexa Fluor-conjugated affinity-purified IgG raised in donkey host (Jackson). Sections were incubated in 300 nM 4′,6-diamidino-2-phenylindole (DAPI) diluted in PBS for 15 min at room temperature. Sections were mounted on SuperFrost Plus slides (Fisher Scientific) using Fluoromount-G (Southern Biotech) and #1.5 cover slips.

### Imaging

Adult brain tissue was imaged on a CSU-W1 spinning disk confocal microscope (Yokogawa). For the initial WT, α-KO, and β-KO imaging, 30-μm Z-stacks were acquired at 2-μm Z-intervals using the ×20 objective. Subsequent images were 20-μm Z-stacks acquired at 2-μm Z-intervals using the ×10 objective. This was done to save on data storage and acquisition time but did not affect the analysis (see Semi-Automated Cell Quantification). Image fields were tiled and stitched in NIS-Elements (Nikon). Perinatal brain tissue was imaged on a Zeiss Axio Imager M2 epifluorescence microscope. In adult and perinatal cases, at least 3 images of comparable regions of interest (ROIs) were captured per mouse.

### Semi-Automated Cell Quantification

Raw images were processed for quantification using a series of custom ImageJ macro scripts. To blind the experimenter to the genotype of the mouse, images were assigned a random code using an existing ImageJ macro script (Filename Randomizer, Tiago Ferreira, 2009) at the time the image file was saved. The experimenter was unblinded after data analysis was complete. Median intensity projection images were generated (ImageJ) from 4 consecutive optical slices of each stack to generate an 8-μm optical section that minimized unwanted signal from cells located deeper in the tissue outside of the focal plane. Tiled images of sagittal sections were assigned an “Allen Brain Atlas Number” from 1 to 21 based on the P56 Sagittal Reference Atlas (Allen Institute). Based on atlas number and stereotaxic coordinates Franklin and Paxinos 2013. The mouse brain in stereotaxic coordinates), images containing specific functional ROI such as V1 and somatosensory cortex were identified for analysis. Multichannel images were split into single channels. DAPI was used to manually specify layers drawn onto the image using ImageJ selection tools, on an ROI roughly 1 mm in width. L1 was not included in the analysis because PV and SST interneurons are absent in this layer. Cortical layers were visually distinguishable from each other based on differences in density of DAPI-stained nuclei. Line selections were manually drawn onto the image to demarcate the boundaries between each layer. The *xy* coordinates of the area and line selections were stored.

Single-channel images containing cells were processed with a custom ImageJ pipeline incorporating standard image filtering and automatic thresholding tools built into ImageJ. A difference of Gaussians filter was applied to the image. This filter applies a Gaussian blur to the original image, with σ_1_ slightly smaller than the smallest cells in the image. Then, a second Gaussian blur with σ_2_ = 2*σ_1_ was applied to the original image. The resulting blurred image is subtracted from the first less blurred image, and results in an image where desired large bright objects, such as cells, are enhanced, whereas undesired small bright objects, such as fine staining of PV-positive neuronal projections, are suppressed. In order to correct brightness variability across the image, we applied contrast-limited adaptive histogram equalization. Finally, we applied the default automatic thresholding algorithm built into ImageJ to obtain a binarized version of the original image. Then, we processed the binarized image with a watershed algorithm and selected particles based on size and circularity to obtain a final set of automated cell counts. A final manual quality control step was performed to ensure cells were counted accurately. After this step, the *xy* coordinates of PV and SST cells were stored.

### Spatial Statistics

Once all *xy* coordinates of regions, layers, and cells were obtained, spatial measurements such as cortical thickness, cell density, nearest neighbor distance (NND), variance of the NND, and paired correlation function (PCF) were computed using Spatstat (Baddeley and Turner 2005), an open-source platform for analyzing spatial point patterns in R.

#### Cortical Dimensions

Cortical thickness was calculated for each image by measuring lines spaced at 1-μm intervals along the axis perpendicular to the bottom of layer 6 and calculating the median of the distribution of lines. We generated points at 1-μm intervals along the bottom of layer 6 and then generated the same number of points spread equally along the top of layer 2/3 and calculated the pairwise distance between each pair of points. This same method was used to calculate the thickness of each layer.

#### Cell Density

We calculated cell density by dividing the number of cells counted by the measured area. The fraction of cells in a given layer was determined by calculating the number of cells that were between the layer annotation lines and dividing by the total number of cells in the ROI.

#### Nearest Neighbor Distance

The NND is the distance between a given cell and its closest cell. This calculation was performed between the same cell types as well as different cell types. NND was also used to determine double positive cells. Cells of different types that had NNDs smaller than the measured radius of the cell body were considered double positive.

The variance of the NND can be used to measure the regularity of spacing between cells. Cells with lower variance are more regularly spaced, whereas a higher variance indicates that they are more randomly spaced. In order to account for differences in mean NND that may affect the variance, we divided the standard deviation of the NND by the mean NND to give a dimensionless ratio known as the coefficient of variance (CV). This allowed us to directly compare the regularity in spacing between groups that had different mean NND.

**Figure 1:**
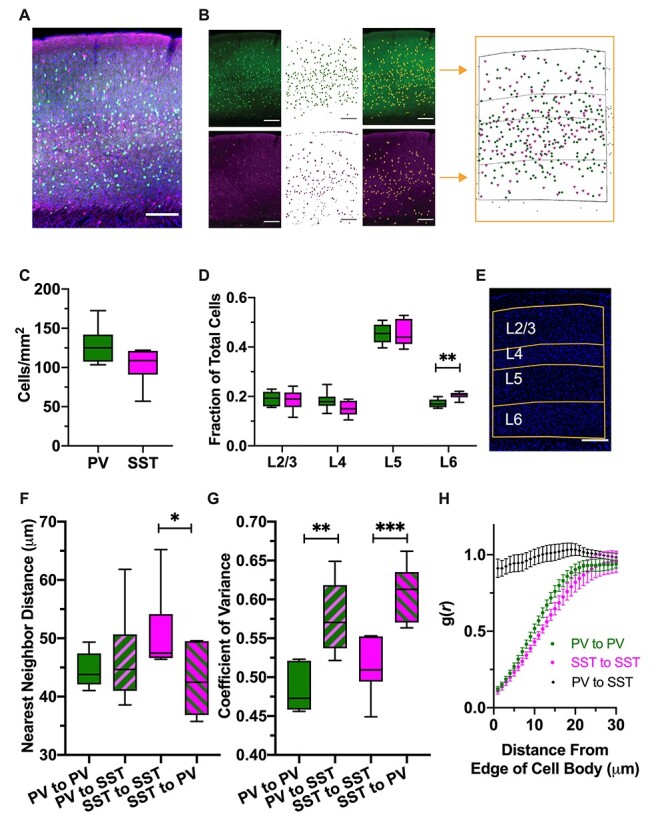
PV- and SST-expressing cINs are spatially independent populations. (*A*) Representative fluorescent image of P30 WT primary visual cortex (V1) labeled with DAPI (blue), anti-PV(green), and anti-SST (magenta), with cortical layers obtained from DAPI image (yellow); scale bar = 200 μm. (*B*) Raw images of PV (left top) and SST (left bottom) are processed to obtain *xy* coordinates of the cells (far right). See Supplementary Figure 1 for more detail. (*C*) Cell density (cells/mm2) of PV and SST neurons. PV neurons tend to be denser than SST neurons (*P* = 0.0629, unpaired *t*-test). (*D*) Relative proportion of PV and SST neurons in each cortical layer in V1, WT. PV and SST neurons are distributed similarly, but there are relatively more SST neurons in L6 (left to right [LtR]: *P* = 0.9965, 0.3474, 0.999, 0.0095, 2-way RM-ANOVA with Sidak’s multiple comparisons test [SMCT]). (*E*) Representative image of DAPI-stained (blue) tissue with layers of the cortex marked (yellow). (*F*) NND between PV–PV, PV–SST, SST–SST, and SST–PV pairs in V1, WT. NND was not significantly different between PV–PV and PV–SST (*P* = 0.2969, Wilcoxon signed-rank test), but SST–SST pairs are significantly more distant than SST–PV pairs (*P* = 0.0424, paired *t*-test). (*G*) CV of the NND for PV–PV, PV–SST, SST–SST, and SST–PV pairs in V1, WT. CV was significantly lower between PV–PV pairs compared with PV–SST pairs (*P* = 0.0013, paired *t*-test) and SST–SST pairs compared with SST–PV pairs (*P* = 0.0002, paired *t*-test). (*H*) PCF of PV–PV pairs (green), SST–SST pairs (magenta), and PV–SST pairs (black). Distance between PV–SST pairs is random, but distance between like-cell types is statistically nonrandom at relatively small distances (PV–PV: *P* < 0.05 up to 19 μm from edge of cell body, SST–SST: *P* < 0.05 up to 19 μm; 2-way RM-ANOVA with SMCT).

#### Paired Correlation Function

To measure the degree of spatial clustering or inhibition we used the PCF g(*r*), which is the ratio of observed cells at a given distance *r* from a reference cell to the expected cells at distance *r* if the distribution were random. In a completely random distribution, the density of cells at any *r* will be equal to the density of the entire field, so g(*r*) = 1. Values below 1 indicate spatial inhibition, whereas values above 1 indicate spatial clustering. g(*r*) for all cells in an ROI is calculated using kernel smoothing. g(*r*) for each mouse was calculated by averaging each ROI. g(*r*) values for genotypes were the average of the mice.

### Statistics

**Figure 2:**
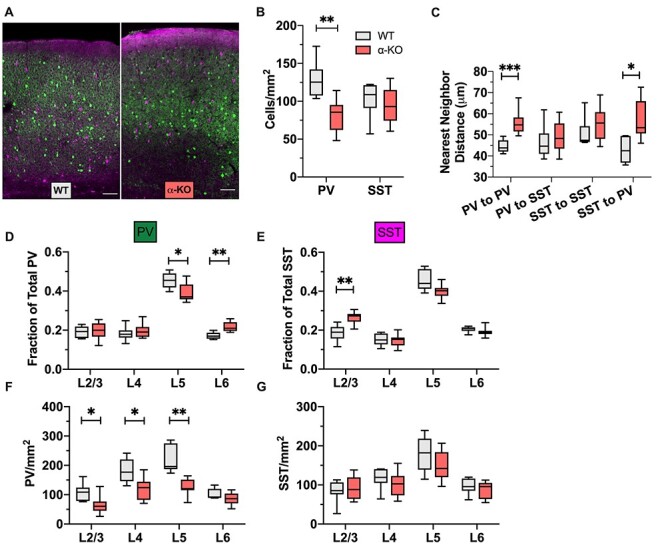
Loss of α-protocadherins reduces the density of PV neurons and alters the laminar distribution of both PV and SST neurons. (*A*) Representative images of WT (left) and α-KO (right) V1, PV (green), and SST (magenta) cIN; scale bar = 200 μm. (*B*) PV and SST cell density in cells/mm2 for WT and α-KO V1 cortex. PV density is reduced, whereas SST density is unchanged (PV: *P* = 0.0033, SST: *P* = 0.7022, 1-way ANOVA with Dunnett’s multiple comparisons test [DMCT]). (*C*) NND between PV–PV, PV–SST, SST–SST, and SST–PV pairs in WT and α-KO V1. NND between PV–PV pairs is increased. The distance between SST–PV pairs is also increased (LtR: *P* = 0.0067, 0.9250, 0.7146, 0.0072, 2-way RM-ANOVA with SMCT). (*D*) Relative proportions of PV cells occupying each cortical layer in V1, WT versus α-KO. The relative amount of PV cells is reduced in L5 and increased in L6 (LtR: *P* = 0.9723, 0.7860, 0.0168, 0.0015, 2-way RM-ANOVA with SMCT). (*E*) Relative proportions of SST cells occupying each cortical layer in V1, WT versus α-KO. The relative amount of SST cells is increased in L2/3 (LtR: *P* = 0.0062, 0.9865, 0.0749, 0.3361, 2-way RM-ANOVA with SMCT). (*F*) Density of PV cells in each cortical layer in V1, WT versus α-KO. The density of PV cells is reduced in all layers except L6 compared with WT (LtR: *P* = 0.0309, 0.0218, 0.0027, 0.2478, 2-way RM-ANOVA with SMCT). (*G*) Density of SST cells in each cortical layer in V1, WT versus α-KO. The laminar density of SST cells is unchanged compared with WT (LtR: *P* = 0.8043, 0.5522, 0.4019, 0.6955, 2-way RM-ANOVA with SMCT).

**Figure 3:**
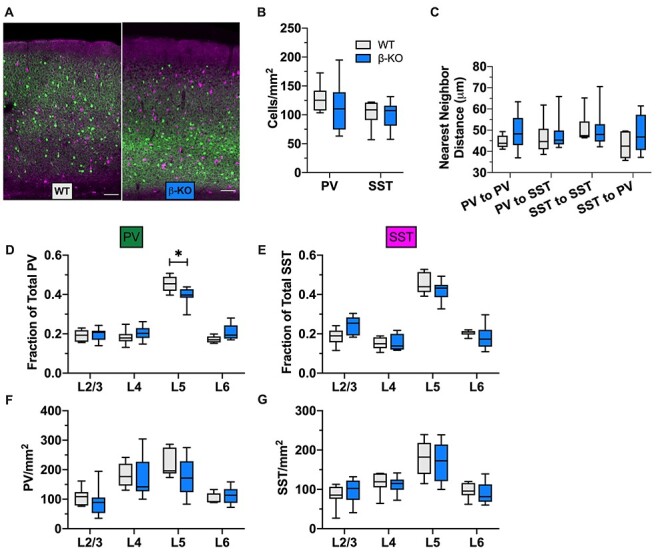
Loss of β-protocadherins alters the laminar distribution of PV cIN. (*A*) Representative images of WT (left) and β-KO (right) V1 PV (green) and SST (magenta) cIN; scale bar = 200 μm. (*B*) PV and SST cell density in cells/mm2 for WT and β-KO V1 cortex. PV and SST density is not different between WT and β-KO (PV: *P* = 0.7932, SST: *P* = 0.9882, 1-way ANOVA with DMCT). (*C*) NND between PV–PV, PV–SST, SST–SST, and SST–PV pairs in WT and β-KO V1. NND was not altered for any group (LtR: *P* = 0.4678, 0.9958, 0.9952, 0.6274, 2-way RM-ANOVA with SMCT). (*D*) Relative proportions of PV cells occupying each cortical layer in V1, WT versus β-KO. The relative amount of L5 PV cells was reduced (LtR: *P* = 0.9925, 0.6148, 0.0386, 0.1312, 2-way RM-ANOVA with SMCT). (*E*) Relative proportions of SST cells occupying each cortical layer in V1, WT versus β-KO. The relative amount of SST cells was unchanged (LtR: *P* = 0.0779, 0.9993, 0.4479, 0.7460, 2-way RM-ANOVA with SMCT). (*F*) Density of PV cells in each cortical layer in V1, WT versus β-KO. The laminar density of PV cells was not changed compared with WT but trended similar to the α-KO in L5 (LtR: *P* = 0.7352, 0.9811, 0.3494, 0.6500, 2-way RM-ANOVA with SMCT). (*G*) Density of SST cells in each cortical layer in V1, WT versus β-KO. The laminar density of SST cells was not changed compared with WT (LtR: *P* = 0.6494, 0.8858, 0.9728, 0.8280, 2-way RM-ANOVA with SMCT).

Statistical tests were calculated in GraphPad Prism 8. We averaged the technical replicates per mouse. Tests and graphs represent these averaged per mouse values. In all cases, QQ plots of residuals were used to check assumptions for normality and used nonparametric alternatives when necessary. We assumed unequal variances and used the Welch *t*-test and Brown–Forsythe 1-way analysis of variance (ANOVA) for all unpaired *t*-tests and 1-way ANOVA tests. For repeated measure (RM) 2-way ANOVA, the Geisser–Greenhouse correction was always applied. Figures 2 and 3 are graphically separated by genotype, but tests were performed on all 3 genotypes together as per the design of the experiment. Full list of statistical tests and *P* values are listed in figure legends. Significance values are reported as following: ^*^P < 0.05, ^**^P < 0.01, ^***^P < 0.001.

## Results

### PV and SST Expressing Interneurons Are Spatially Independent Populations

To characterize the spatial arrangement of cINs in normal and mutant animals, we first established a pipeline to convert raw image data into a form that could be readily analyzed using the spatial analysis software suite, Spatstat (Baddeley and Turner 2005) (Fig. 1*A*,*B*, Supplementary Fig. 1). This method allowed us to analyze a number of different spatial characteristics of cIN classes. For our analysis, we focused on 2 cortical regions in which cINs have been well-studied: primary visual (V1) and primary somatosensory (S1) cortices.

Consistent with existing literature (Rudy et al. 2011), PV neurons were about 20% more dense than SST neurons in V1 (Fig. 1*C*). Both types of neuron were found in every layer besides L1, but the majority of both types are found in L5 (Fig. 1*D*). Both types of neurons have largely similar laminar distribution patterns, but SST neurons are relatively more abundant in L6 compared with L5 (Fig. 1*D*). S1 had no significant differences compared with V1 for all measurements other than cortical thickness, which was generally thicker in S1 (Supplementary Fig. 2).

PV and SST cells share a common developmental origin (Wamsley and Fishell 2017) and largely occupy the same cortical space. We next examined how PV and SST cells are spaced within and between subclasses. The NND between PV–PV pairs was not different than the NND between PV–SST pairs (Fig. 1*F*). SST–SST pairs were significantly more distant compared to SST–PV pairs, which is expected given the lower SST density. To compare the regularity in spacing within and between groups, we used the CV of the NND, which is a normalized measure of variance. A lower CV suggests greater regularity of spacing. The CV was significantly lower in PV–PV pairs compared with PV–SST pairs, as well as for SST–SST pairs compared with SST–PV pairs (Fig. 1*G*). This suggests greater regularity of spacing within groups compared with across groups. To see if cells exhibited spatial inhibition or clustering, we used the PCF, g(*r*) (see Methods-Spatial Statistics-Paired Correlation Function). We found that matching cell type pairs had significantly smaller g(*r*) values than expected at modest distances from their cell bodies, whereas unmatched pairs were not significantly different than random at any distance (Fig. 1*H*). These data suggest that PV cells and SST cells distribute spatially independent of each other but maintain spacing relative to homotypic cells.

### Loss of α-Protocadherins Reduces the Density of PV Neurons and Alters the Laminar Distribution of Both PV and SST Neurons

To study how cPcdhs affect the spatial arrangement of cINs, we performed the same measurements in the context of cPcdh mutants. We studied mice in which either all variable exons of the α-cluster are deleted (α-KO) or all exons of the β-cluster are deleted (β-KO). We did not examine γ-cluster mutants (γ-KO) because they are perinatal lethal (Hasegawa et al*.* 2016; Garrett et al*.* 2019; Mancia Leon et al*.* 2020), before mature cIN spatial distribution is established. Since loss of a cPcdh cluster would reduce the potential pool of cPcdhs any given cell could draw from for self- and cell–cell recognition, we hypothesized that it would result in disruptions to PV and SST cell distribution.

In V1 of the α-KO we found that the density of PV cells was significantly lower compared with WT (Fig. 2*B*). In every layer except L6, PV density was significantly reduced (Fig. 2*F*), whereas the density of SST was unaffected (Fig. 2*B*,*G*). The NND between PV pairs was also significantly increased in the α-KO (Fig. 2*C*), consistent with a reduction in cell density. Indeed, we found that the NND was significantly correlated with cell density for all cell types and genotypes analyzed (Supplementary Fig. 3). We also noted a change in the relative distribution of PV cells, with relatively fewer PV cells in L5 and more PV cells in L6. (Fig. 2*D*). With SST cells (Fig. 2*B*,*G*), we noted an increase in L2/3 and a nonsignificant decrease (*P* = 0.0749) in L5 (Fig. 2*E*). In S1, we also found an increase in the relative amount of L6 PV cells, but other measures were unaffected (Supplementary Fig. 4). However, we did note trends that resembled the effects seen in V1 for SST cells, which were just outside the significance threshold (Supplementary Fig. 4*E*). Altered laminar distribution in both PV and SST were not accompanied by changes in CV or PCF (Supplementary Fig. 5).

Differences in relative layer thickness between WT and α-KO were a potential confound. To assess this, we quantified cortical thickness, as well as the thickness of its layers. The cortex of the α-KO was significantly thicker overall and in L2/3 and L6 (Supplementary Fig. 6*A*,*B*). However, layers were proportionally similar to WT (Supplementary Fig. 6*C*), indicating that changes associated with L5 or L6 were not the result of the layers themselves being altered. We did find that L2/3 was modestly, but significantly greater (2%) as a relative portion of the α-KO cortex (Supplementary Fig. 6*C*). In contrast, the difference in relative proportion of SST cells in L2/3 (19% in WT vs. 27% of SST in α-KO) was robust, suggesting that the change in relative cortical thickness in α-KO would be at most, a minor contributing factor.

### Loss of β-Protocadherins Alters the Laminar Distribution of PV Interneurons

Like the α-KO, we observed a significant reduction in the relative amount of PV cells in L5 (Fig. 3*D*) and also an upward trend in L6 (*P* = 0.1312). PV laminar density was mostly unchanged (Fig. 3*B*,*F*). The mean density of SST cells was unchanged overall or in any layer compared with WT (Fig. 3*B*,*G*). We observed an upward trend in the relative amount of SST cells in L2/3 (*P* = 0.0779, Fig. 3*E*). Together, our analysis of the β-KO revealed a similar, but milder, phenotype compared with α-KO.

### The Observed Adult α-KO Phenotype Is Unlikely to Be Due to Increased Repulsion During cIN Radial Migration or Early Perinatal Cell Death

A series of 3 stages of cIN development occur during perinatal time points that could potentially influence cIN spatial distribution: 1) radial migration, which occurs from P2 to P5; 2) programmed cell death, which occurs from P4 to P12 and peaks at P7 (Southwell et al*.* 2012); 3) synaptogenesis, which occurs between P8 and P16 (Favuzzi et al. 2019). Given that the spatial distribution phenotypes observed in α-KO and β-KO were similar but milder in β-KO, we focused on the α-KO for our analysis during perinatal time points: P3, P7, and P13.

**Figure 4:**
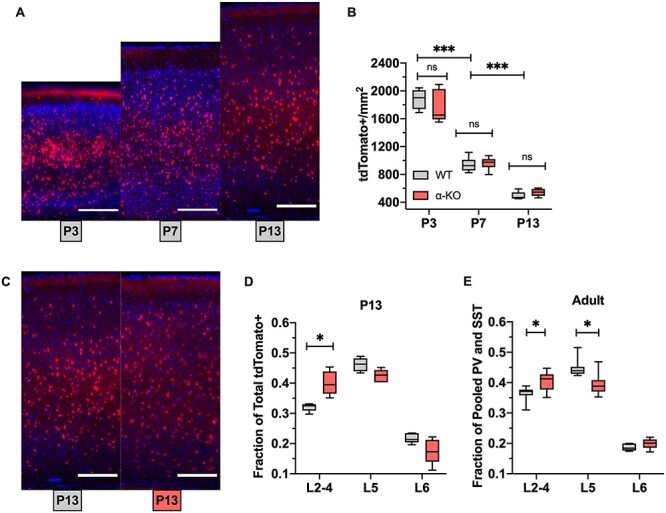
cIN laminar distribution similar to the adult α-KO V1 emerges at P13. (*A*) Representative images of tdTomato+ (red) neurons counterstained with DAPI (blue) from Nkx2.1Cre; Ai9 V1 at (LtR:) P3, P7, P13; scale bar = 200 μm. (*B*) Density of tdTomato+ cells in V1 at P3, P7, and P13, Nkx2.1Cre; Ai9; cPcdhWT versus α-KO. The density of tdTomato+ cells decreases over time but does not differ between WT and α-KO (WT vs. α-KO: P3 *P* = 0.7789, P7 *P* = 0.725, P13 *P* = 0.4758, unpaired *t*-tests; *P* < 0.0001 for comparisons between time points, 2-way ANOVA with SMCT). (*C*) Representative images of tdTomato+ (red) neurons counterstained with DAPI (blue), from P13 Nkx2.1Cre; Ai9; cPcdhWT (left) or Nkx2.1Cre; Ai9; α-KO (right) V1; scale bar = 200 μm. (*D*) Relative proportion of tdTomato+ cells in each cortical layer in V1 at P13, Nkx2.1Cre; Ai9; cPcdhWT versus α-KO. The proportion of superficial tdTomato+ cells is increased in the α-KO at P13 (L2–4: *P* = 0.0245, L5: *P* = 0.0779, L6: *P* = 0.2353, 2-way RM-ANOVA with SMCT). (*E*) Relative proportion of pooled PV and SST cells in each cortical layer from V1 P30 α-KO. Proportion in superficial layers is increased, whereas L5 is decreased, similar to the P13 Nkx2.1Cre; Ai9; α-KO (L2–4: *P* = 0.0282, L5: *P* = 0.0129, L6: *P* = 0.1683, 2-way RM-ANOVA with SMCT).

Interneuron migration in the context of PV and SST cells is difficult to directly study, due to the relatively late expression of PV (Vogt Weisenhorn et al. 1998). Thus, to visualize cells early on, we made use of Nkx2.1^Cre^; Ai9 mice, which label all MGE-derived cINs with tdTomato (Fig. 4*A*). This strategy allowed us to study the spatial characteristics of a combined population of PV and SST cells in the context of cPcdh mutations and to infer the timing for when the adult mutant phenotype emerges perinatally.

Consistent with prior reports (Southwell et al*.* 2012), the density of tdTomato+ cells decreases between P3 and P13 (Fig. 4*B*), with no differences between WT and α-KO (Supplementary Fig. 7*A*). This coincides with an expansion in cortical thickness during the same time frame (Supplementary Fig. 7*A*). At P3 and P7, we observe no changes in the relative laminar distribution of tdTomato+ cells (Supplementary Fig. 7*C*,*D*). However, at P13, there is a significant increase in the fraction of tdTomato+ cells in upper layers (Fig. 4*C*,*D*) and a trend toward reduced L5 (*P* = 0.0779). In the perinatal datasets, it was difficult to distinguish L4 from L2/3 using DAPI, but the border between L4 and L5 was always clear. To compare our results with the adult α-KO results, we pooled PV and SST, as they encompass the entire Nkx2.1 lineage in the cortex (Rudy et al*.* 2011). We also pooled L2/3 and L4 since the distinction between these layers is not clear at early perinatal time points. With the caveat that IHC labeling is not exactly the same as genetic fate mapping, we found that the laminar distribution in the adult α-KO was similar to that of the P13 Nkx2.1Cre; Ai9; α-KO (Fig. 4*E*).

### Loss of a Single Protocadherin, pcdh-αc2, Alters the Laminar Distribution of SST Cells but Does Not Change Cell Density

Our data indicate that the loss of cPcdhs differentially affects PV and SST neurons, yet the majority of cPcdh isoforms are expressed stochastically in most if not all cortical neurons (Chen and Maniatis 2013). The exceptions are pcdh-αc1, αc2, γc3, γc4, and γc5, which are transcriptionally regulated (Kawaguchi et al. 2008; Chen and Maniatis 2013). Although these regulated isoforms are broadly expressed, single-cell RNA-sequencing data from the Allen Brain Institute (Tasic et al. 2018) suggests that certain classes of cells have enriched expression. Pcdh-αc2 is highly expressed in PV cells, and to a lesser extent SST, in adult V1 (Supplementary Fig. 8). Based on these data, we explored the contribution of pcdh-αc2 to the α-KO phenotype using a pcdh-αc2-specific knockout mouse (αc2-KO).

**Figure 5:**
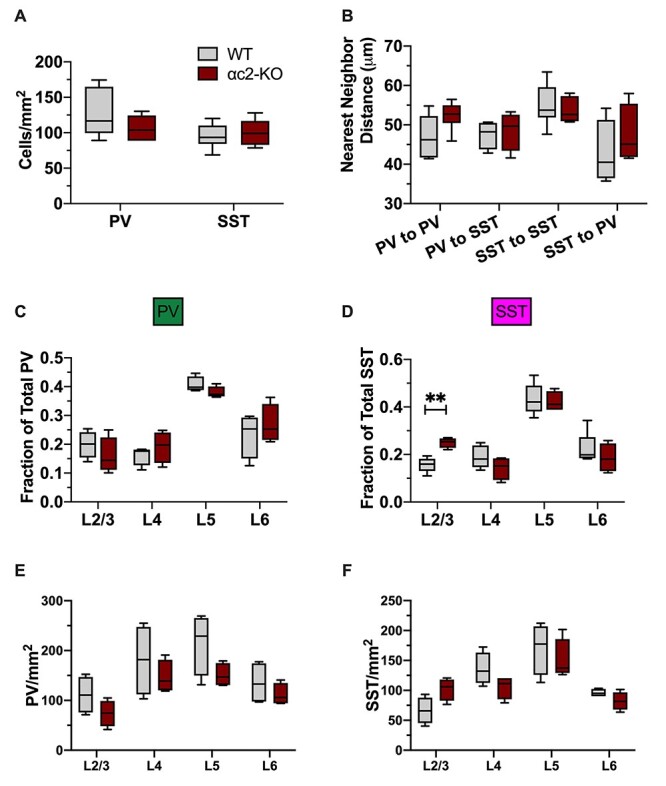
Loss of a single protocadherin, pcdh-αc2, alters the laminar distribution of SST cells but does not change cell density. (*A*) Density of PV and SST cells in V1, WT versus αc2-KO. Cell density is not changed in the αc2-KO (PV: *P* = 0.2313; SST: *P* > 0.9999, unpaired *t*-tests). (*B*) NND between PV–PV, PV–SST, SST–SST, and SST–PV pairs. NND is not changed in the αc2-KO between any group compared (LtR: *P* > 0.9999, 0.9079, 0.8493, 0.6085, 2-way RM-ANOVA with SMCT). (*C*) Relative proportion of PV cells in each cortical layer in V1, WT versus αc2-KO. The relative proportion of PV cells was not changed (LtR: *P* = 0.8338, 0.8728, 0.5121, 0.9374, 2-way RM-ANOVA with SMCT). (*D*) Relative proportion of SST cells in each cortical layer in V1, WT versus αc2-KO. There are relatively more SST cells in L2/3 in the αc2-KO compared with WT but other layers are not altered (LtR: *P* = 0.0044, 0.5831, 0.9981, 0.8895, 2-way RM-ANOVA with SMCT). (*E*) Density of PV cells in each cortical layer in V1, WT versus αc2-KO. PV density is not significantly changed in any layer in the αc2-KO, though L5 does trend lower (LtR: *P* = 0.4938, 0.8987, 0.4221, 0.8515, 2-way RM-ANOVA with SMCT). (*F*) Density of SST cells in each cortical layer in V1, WT versus αc2-KO. SST density is not significantly changed in any layer in the αc2-KO, though it does trend up in L2/3 (LtR: *P* = 0.1815, 0.4139, 0.9420, 0.5415, 2-way RM-ANOVA with SMCT).

While pcdh-αc2 is enriched in adult PV cells, we did not observe significant changes to PV density or NND in αc2-KO mice (Fig. 5*A*,*B*). Laminar distribution was also unchanged for PV cells (Fig. 5*C*). With SST cells, however, we found that the αc2-KO recapitulated some aspects of the α-KO phenotype: a significant increase in the relative amount of SST cells in L2/3 (Fig. 5*D*) without a change in SST density or NND (Fig. 5*A*,*B*). Unlike the α-KO, we did not see a significant reduction in L5 (Fig. 5*D*). We also did not observe a change in cortical thickness (Supplementary Fig. 9*A*–*C*) or CV (Supplementary Fig. 9*D*) in the αc2-KO. Together these data suggest that pcdh-αc2 may be playing a larger role in SST cell layer distribution compared with PV.

## Discussion

The spatial positioning of neurons can be critical to their function, as is the case in sensory neurons in *Drosophila* (Matthews et al. 2007; Liu et al. 2020) or starburst amacrine cells in retina (Fuerst et al*.* 2008; Garrett et al. 2018; Ing-Esteves et al. 2018). In this study, we analyzed the spatial position of cINs in WT and cPcdh mutants. cINs originate from distal progenitor zones and distribute throughout the cortex during development through extensive migration and cell death (Tremblay et al*.* 2016; Wamsley and Fishell 2017). We hypothesized that cIN subclasses self-recognize during development to ensure that subclass-specific circuits are evenly distributed. We also considered the possibility that generic repulsion might exist between all cINs to further facilitate even distribution of inhibitory networks. In our detailed analysis of cIN spatial distribution in the WT, however, PV and SST cells appear to ignore the presence of the other cell type even though both cells occupy similar laminar domains. This suggests that generic interneuron–interneuron repulsion does not occur. Rather, our data indicate that cell–cell repulsion only exists within cIN subclasses and not between them. This is reminiscent of the retina in which bNOS- and dopaminergic amacrine cell subtypes are patterned independently of heterotypic cells but are mostly nonrandomly spaced with respect to homotypic cells (Fuerst et al*.* 2008).

Our examination of cPcdh mutants revealed cIN subclass-specific alterations in spatial distribution, cell density, and laminar distribution. For instance, we observed that PV density was significantly reduced in the α-KO, whereas SST density was unchanged. PV density reduction was accompanied by a significant increase in PV cell NND. A recent study reported that the γ-cluster (but not α- or β-clusters) is important for cIN survival (Mancia Leon et al*.* 2020). It is important to note that in this study, the authors quantified Nkx2.1^Cre^ fate-mapped neurons without distinguishing between PV and SST in their analysis of α-KO and β-KO mice. Thus, the decrease in PV density we observed may have been masked by quantifying the entire Nkx2.1 (MGE) lineage as whole. Interestingly, however, Mancia and colleagues note trends toward decreased Nkx2.1 lineage cell density, consistent with our findings. Further, in the spinal cord γ-cluster cPcdhs generally mediate interneuron survival, but FoxP2+ subtypes of spinal cord interneurons are reduced in both α- and β-cluster mutants (Hasegawa et al*.* 2016). While the mechanism driving the decrease in PV density we observe is unclear, α-KO PV cells exhibit connectivity defects including reduced inhibitory synapses, reduced neurite number, and reduced neurite length (Shao et al. 2019). As such, alterations in connectivity may underlie increased PV cell death, resulting in decreased cell density. We also observed subtype-specific changes in the relative laminar distribution of V1 α-KO cINs. Both PV and SST cells were reduced in L5, but PV cells were increased in L6, whereas SST cells were increased in L2/3. We noted similar shifts in laminar distribution in S1 and in the β-KO, although some of these effects were trends rather than significant changes.

While we observed clear subtype-specific phenotypes in laminar distribution and cell density in cPcdh mutants, most cPcdh isoforms are stochastically expressed (Chen and Maniatis 2013). To reconcile this, we examined existing single-cell RNA-sequencing datasets of adult mouse visual cortex (Tasic et al*.* 2018) and found that a nonstochastic member (Kawaguchi et al*.* 2008; Chen and Maniatis 2013) of the α-cPcdhs, pcdh-αc2 was highly expressed in subgroups of PV and SST cells. When we examined spatial distribution of PV and SST in αc2-KO mice, we found that L2/3 SST cells were increased, whereas SST cells in other layers and all PV cells were mostly unaffected. This finding supports the notion that nonstochastic subtype-specific expression of cPcdhs helps regulate cIN distribution. While it is not clear why SST cells are specifically affected, it is possible that pcdh-αc2 expression is enriched early in SST cells, whereas enriched PV expression occurs after the mutant phenotype emerges (P13).

Taken together, we observed subclass-specific laminar distribution phenotypes that were strikingly similar across 3 different cPcdh mutants and in 2 different cortical areas (V1 and S1). Loss of cPcdh diversity can aberrantly increase recognition and repulsion between neurons that do not normally repel (Lefebvre et al*.* 2012). Further, shortly after birth, interneurons undergo radial migration into the cortical plate to settle in their final layer (Marin 2013). Thus, an appealing model is that aberrant cPcdh matching due to decreased cPcdh diversity would in turn alter subclass spacing. Here, cIN radial migration would be an ideal process for cPcdh regulation since laminar position is largely determined in this phase. However, when we examined α-KO mice during perinatal time points, we did not observe changes in laminar distribution until P13, well after radial migration has ceased.

The precise mechanism for how loss of cPcdh diversity alters cIN subclass spacing remains to be determined. One possibility is cell intrinsic: Previous studies have found that α-KO PV cells possess morphological defects and reduced inhibitory synapse density, whereas excitatory synapse density was not changed (Shao et al*.* 2019), suggesting defects in intrinsic connectivity. Another possibility, perhaps acting in parallel, is that loss of cPcdh diversity increases the likelihood of neighboring cINs to mistakenly identifying nonself neurites as self neurites, as has been observed in retina (Lefebvre et al*.* 2012). In the cortex, PV and SST cells occupy similar spatial domains and densely populate deep layers, in particular L5. cPcdh diversity could therefore enable cINs of the same subclass to pack into high-density areas such as deep layers of the cortex. cIN target innervation is similarly dense, with many cINs targeting the same PC, and many PCs being inhibited by the same cIN (Fino and Yuste 2011; Packer et al*.* 2013). Both cell intrinsic and extrinsic defects could then lead to altered connectivity and reduced activity, which would reduce the number of cINs (Priya et al. 2018) in specific areas, and most prominently in areas where cINs are most dense. This would potentially account for why the phenotype in L5 of V1 was more prominent—V1 is significantly thinner than S1, which would place even more spatial constraint on cINs and increase the chances of encountering an aberrantly matched cIN. This possibility could be directly tested in future studies by reconstructions of interneuron morphology in cPcdh mutants.

Alternatively, alterations in cIN laminar distribution could be due to selective survival during development as a result of alterations is cortical activity. cIN survival is regulated by neuronal activity (Priya et al*.* 2018; Wong et al. 2018), and aberrant neuronal activity can alter the laminar positioning in cINs of the caudal ganglionic eminence (De Marco Garcia et al. 2011). Deficits in serotonergic wiring have also been reported in the α-KO and αc1/2-KO mice (Katori et al. 2009; Chen et al*.* 2017) and alterations in serotonergic tone could also affect overall interneuron activity. Interestingly, deficits in visual acuity and aggregation of retinogeniculate terminals have been reported to appear between P10 and P14 in the α-KO (Meguro et al. 2015), coinciding with the emergence of cIN phenotype we observed at P13. This could alter the activity state of the visual cortex, where we observe the most robust mutant phenotype.

cINs function by establishing a distributed network of locally projecting neurons throughout the cortex. Our study indicates that while cPcdhs do not affect subclass cell–cell repulsion, they play an important role in regulating how cINs spatially distribute among cortical layers. The findings also demonstrate that cPcdh loss of function differentially affects interneuron subtypes. Indeed, specific genetic ablation of αc2 largely phenocopies the mutant SST phenotype of the whole cluster α-KO, whereas PV cells are unaffected. This suggests that nonstochastic cPcdh expression helps establish subtype-specific spatial distribution patterns, possibly through subtype-specific expression. Finally, when we profiled cIN spatial arrangement during perinatal time points, we found that the α-KO phenotype only emerged later at P13, well after radial migration, and coinciding with cIN morphological elaboration and synaptogenesis. Thus, our work raises the intriguing possibility that cPcdh isoforms, expressed with subtype specificity, govern early circuit integration and thereby differential cIN cell survival. It remains to be determined whether this is the result of cell-autonomous or circuit-level mechanism. Previous findings suggest both could be at play and that both could serve as compelling avenues to pursue for future studies.

## Supplementary Material

sup_figs_revised_EA_tgaa089

## Notes

The authors would like to thank Tom Maniatis for generously providing cPcdh-mutant animals as well as Chiamaka Nwakeze and Amy Kirner for their help with these animals. Thanks to Luke Hammond and Tu Nguyen for their help with imaging and Kevin Sitek for help with coding. Also thanks to Wes Grueber, Carol Mason, Franck Polleux, Richard Vallee, and Hynek Wichterle for thoughtful comments on the project and the manuscript. Finally thank you to Luke Nunnelly, Patrick Dummer, Sofia Leal Santos, and Melissa McKenzie for their feedback and discussion. *Conflict of Interest*: None declared.
