# Supplementary material for: Loss of Clustered Protocadherin Diversity Alters the Spatial Distribution of Cortical Interneurons in Mice
Source: Cereb Cortex Commun. 2020 Nov 25;1(1):tgaa089. doi: 10.1093/texcom/tgaa089 (PMC8152951; doi:10.1093/texcom/tgaa089)
Supplement: sup_figs_revised_EA_tgaa089 [file sup_figs_revised_ea_tgaa089.zip › sup_figs_revised_EA_tgaa089.pdf]

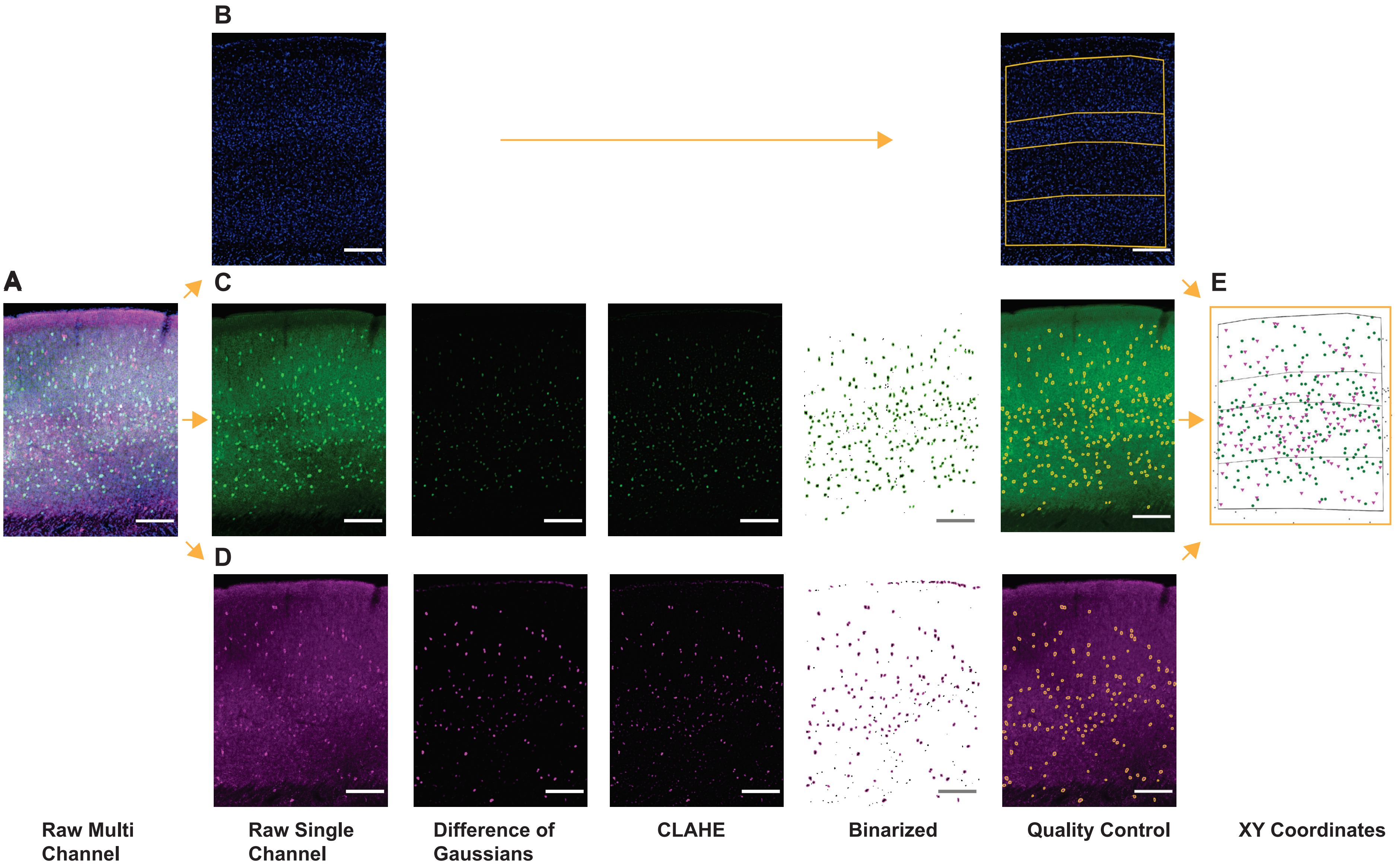

Supplemental Figure 1

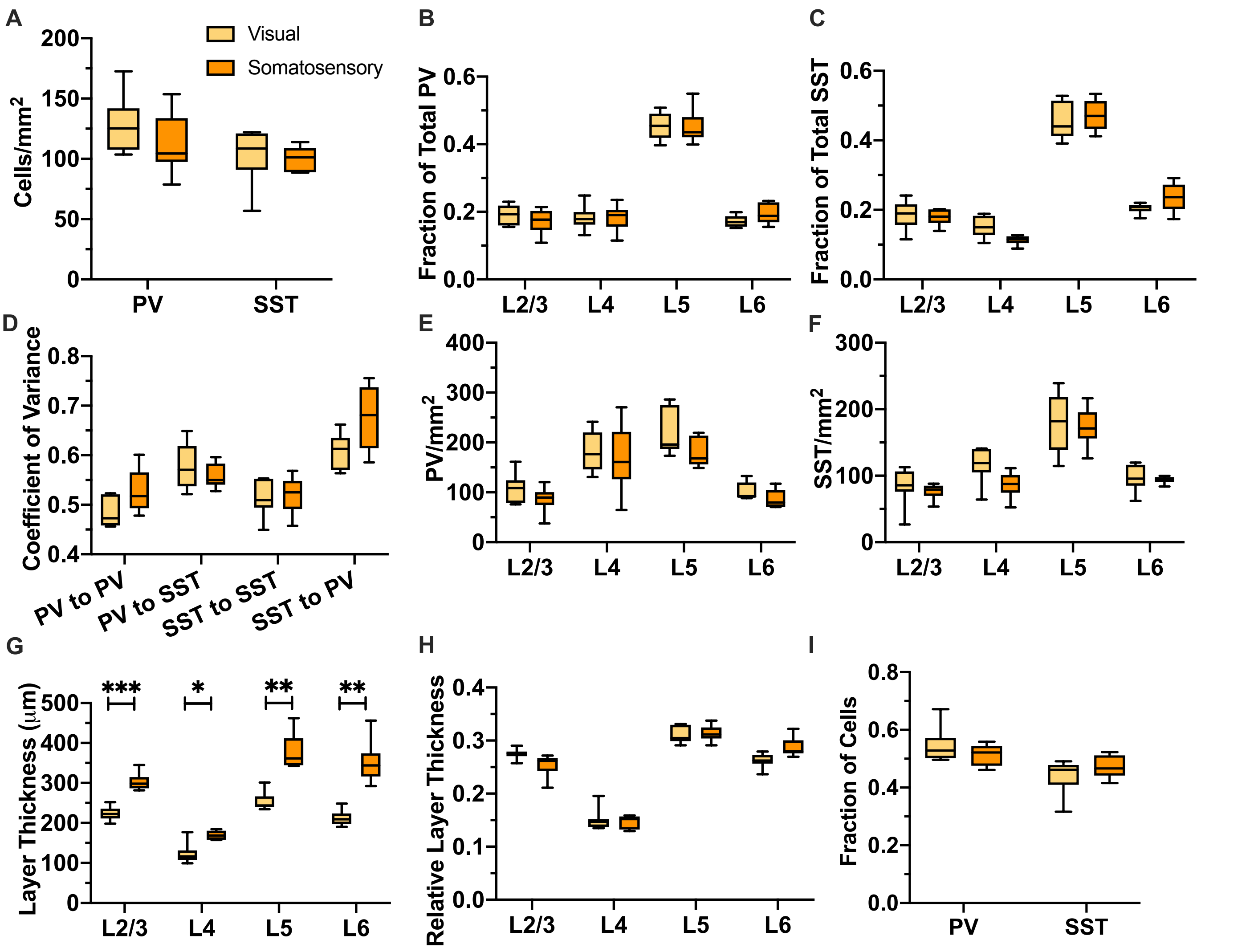

Supplemental Figure 2

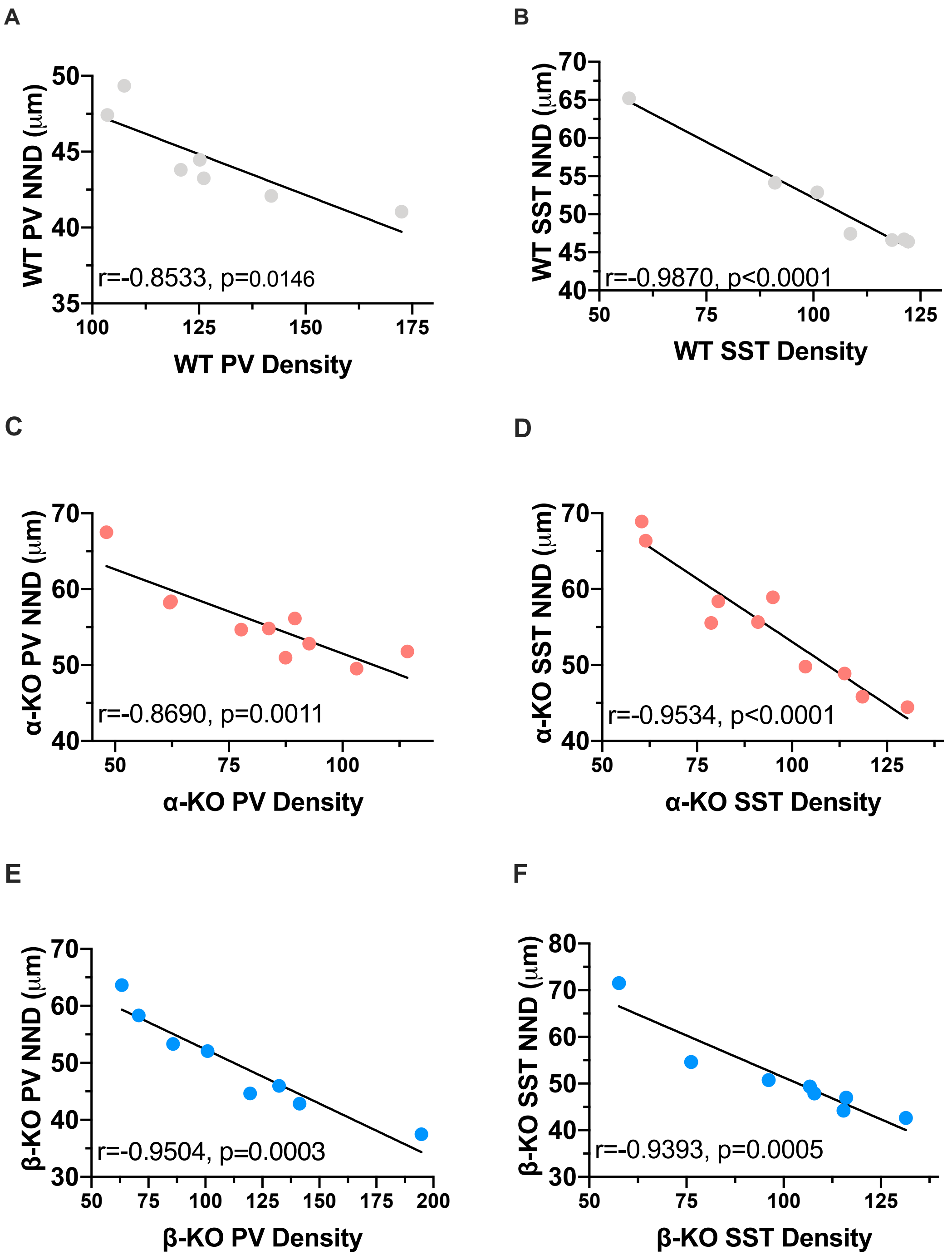

Supplemental Figure 3

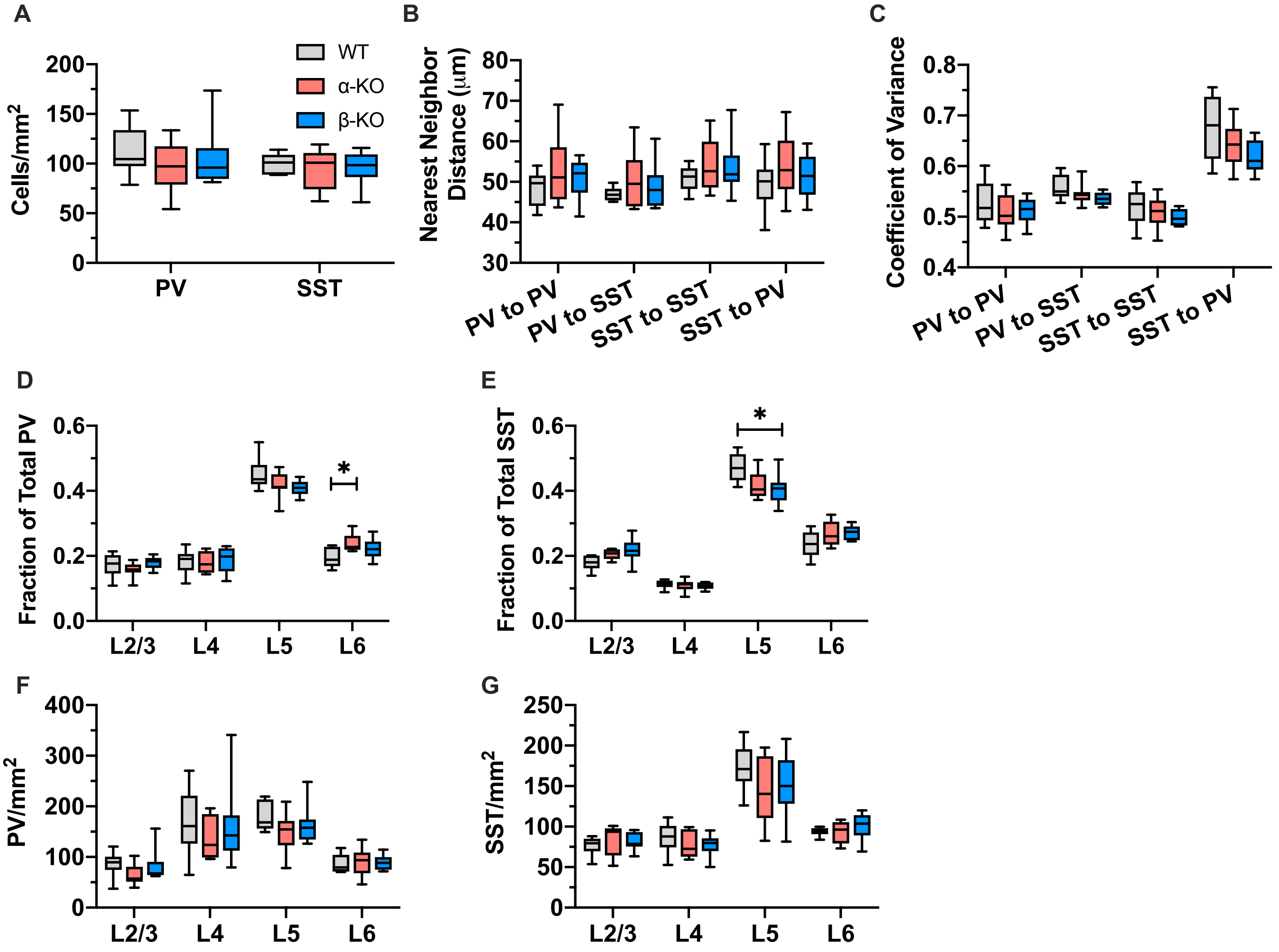

Supplemental Figure 4

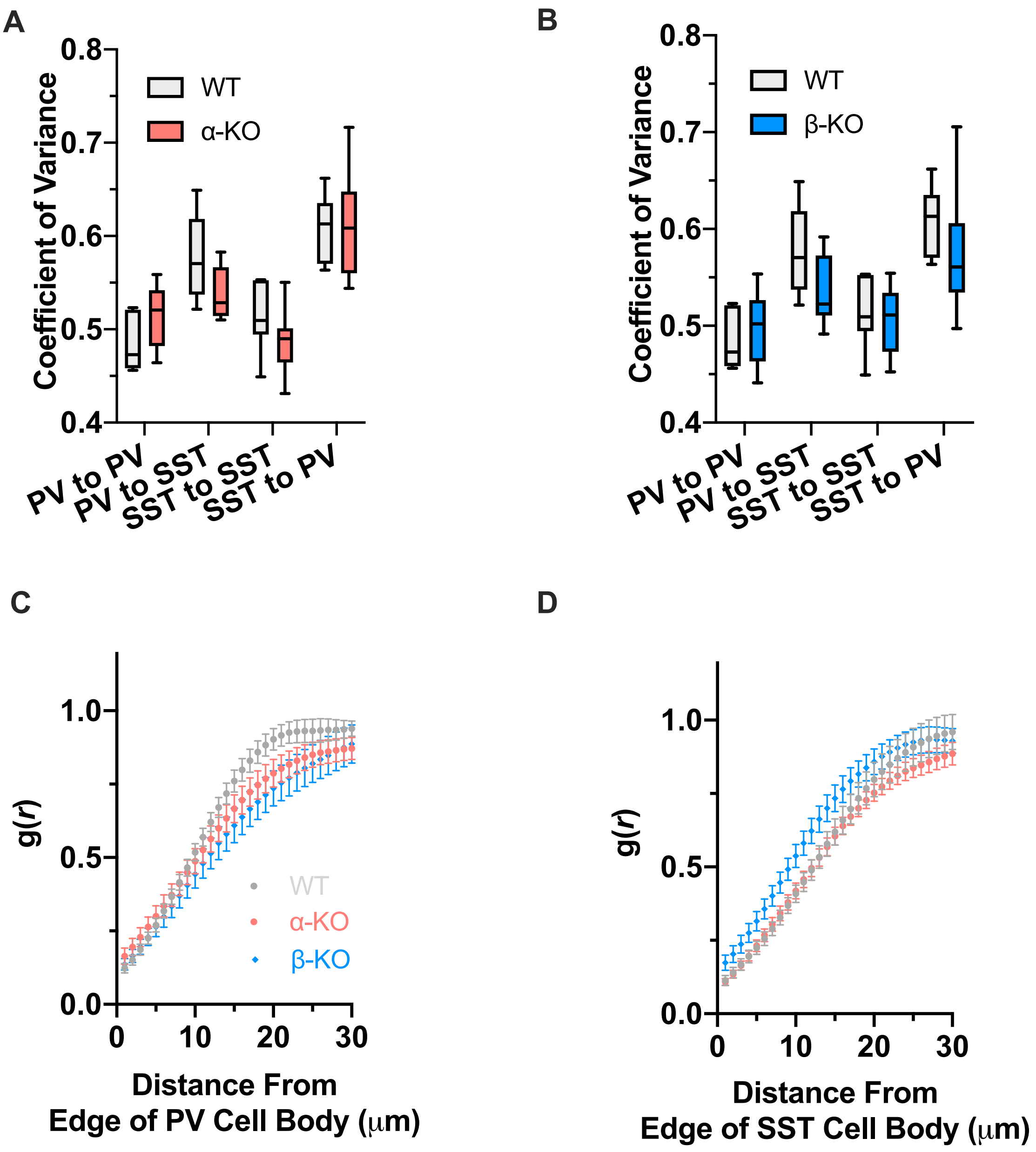

Supplemental Figure 5

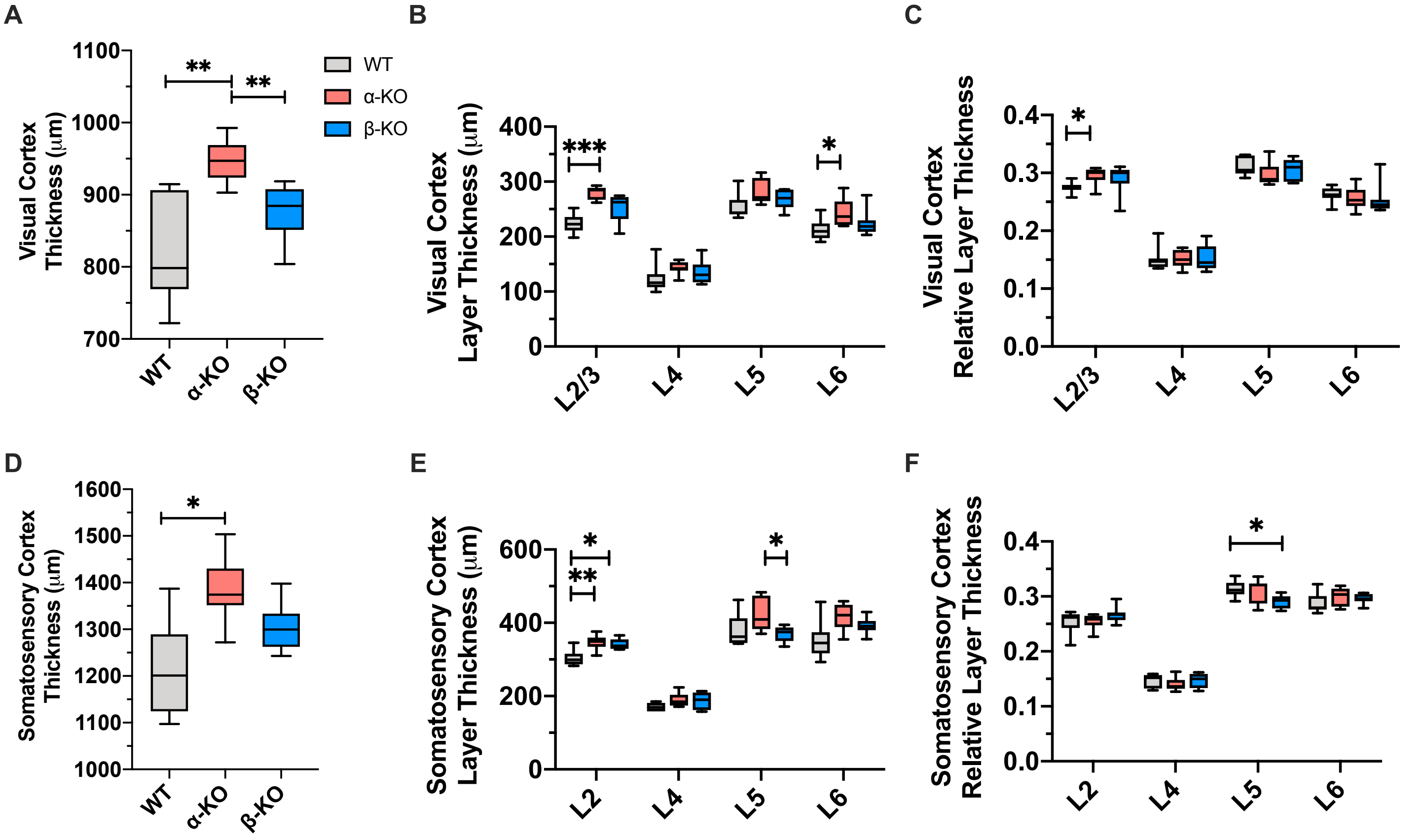

Supplemental Figure 6

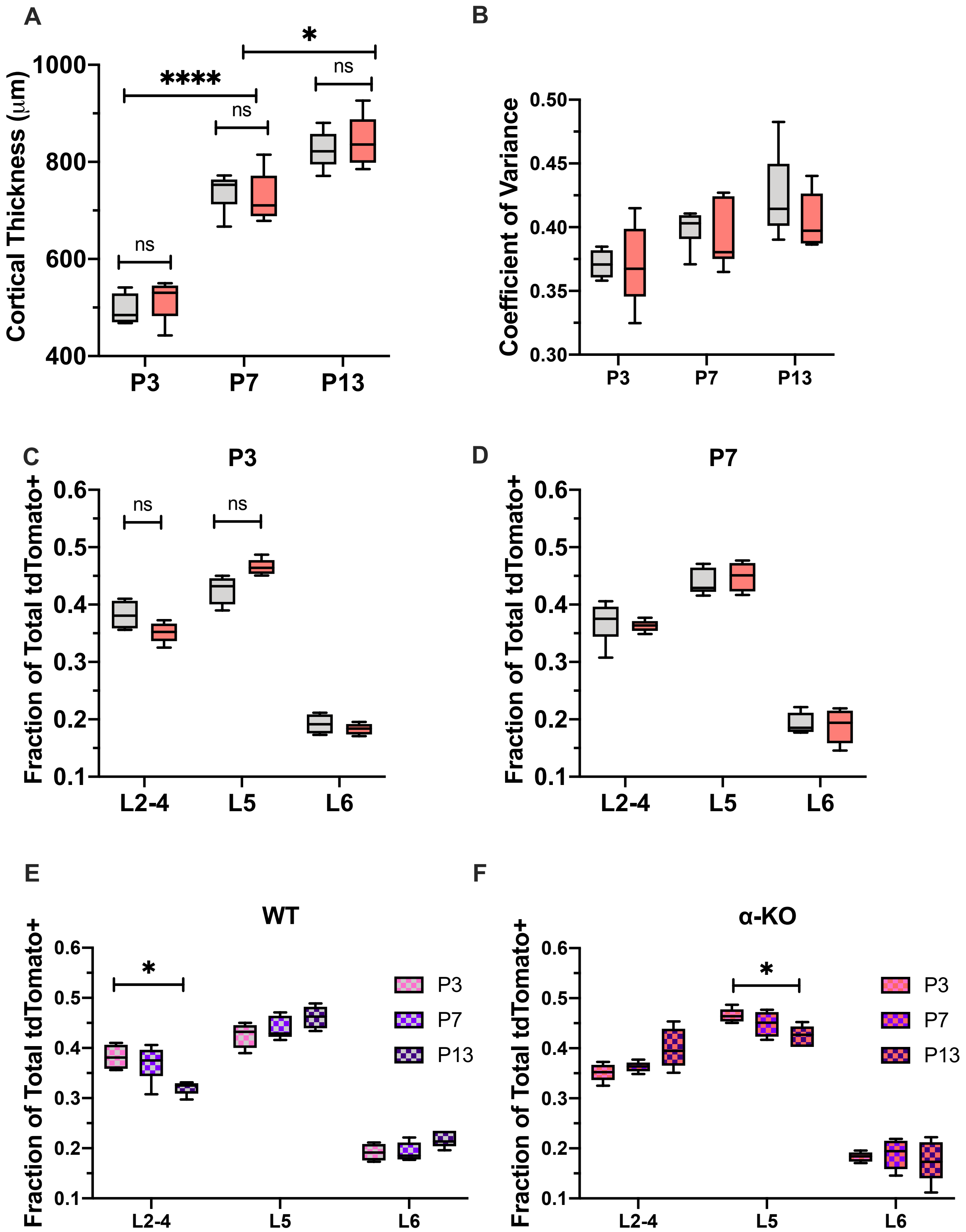

Supplemental Figure 7

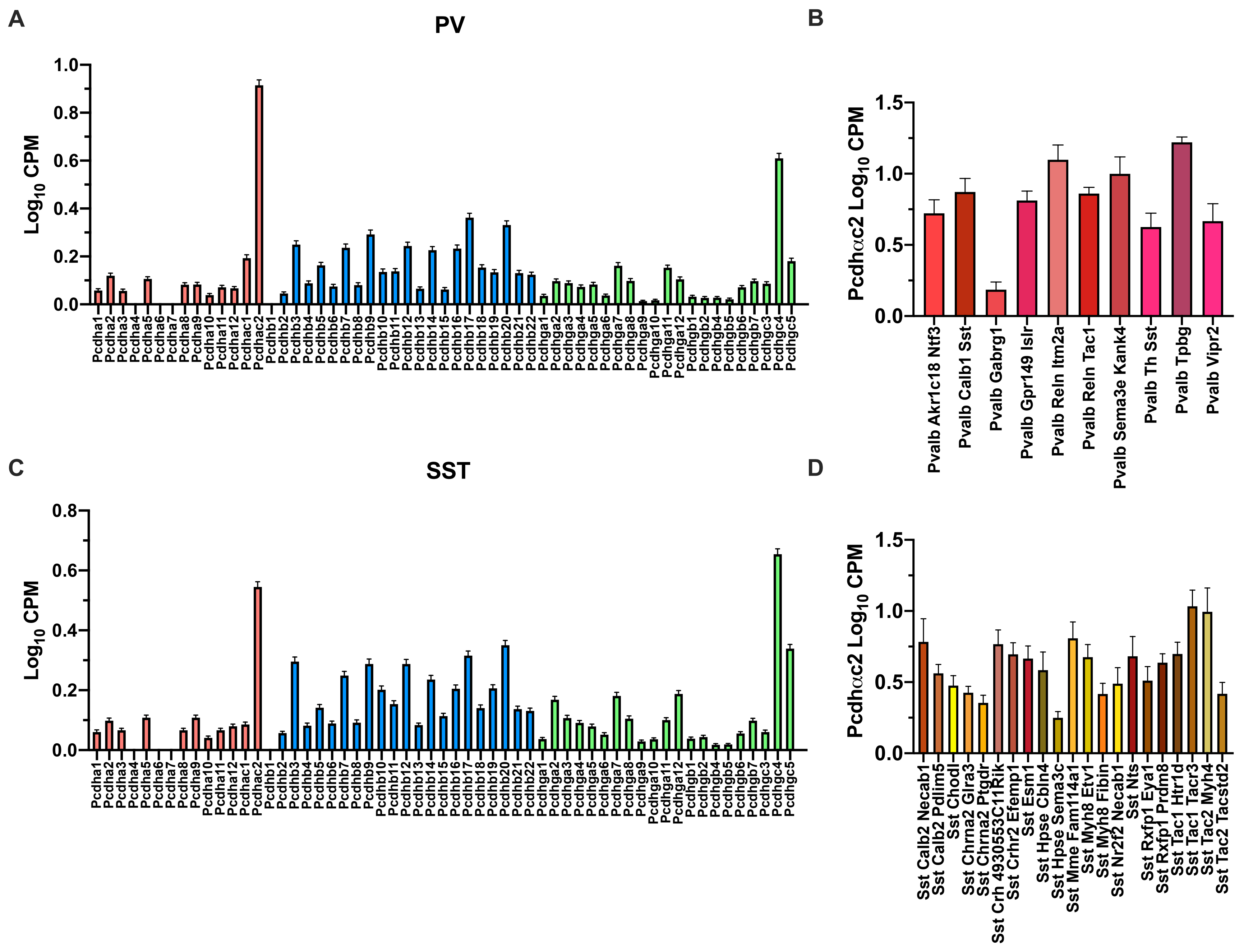

Supplemental Figure 8

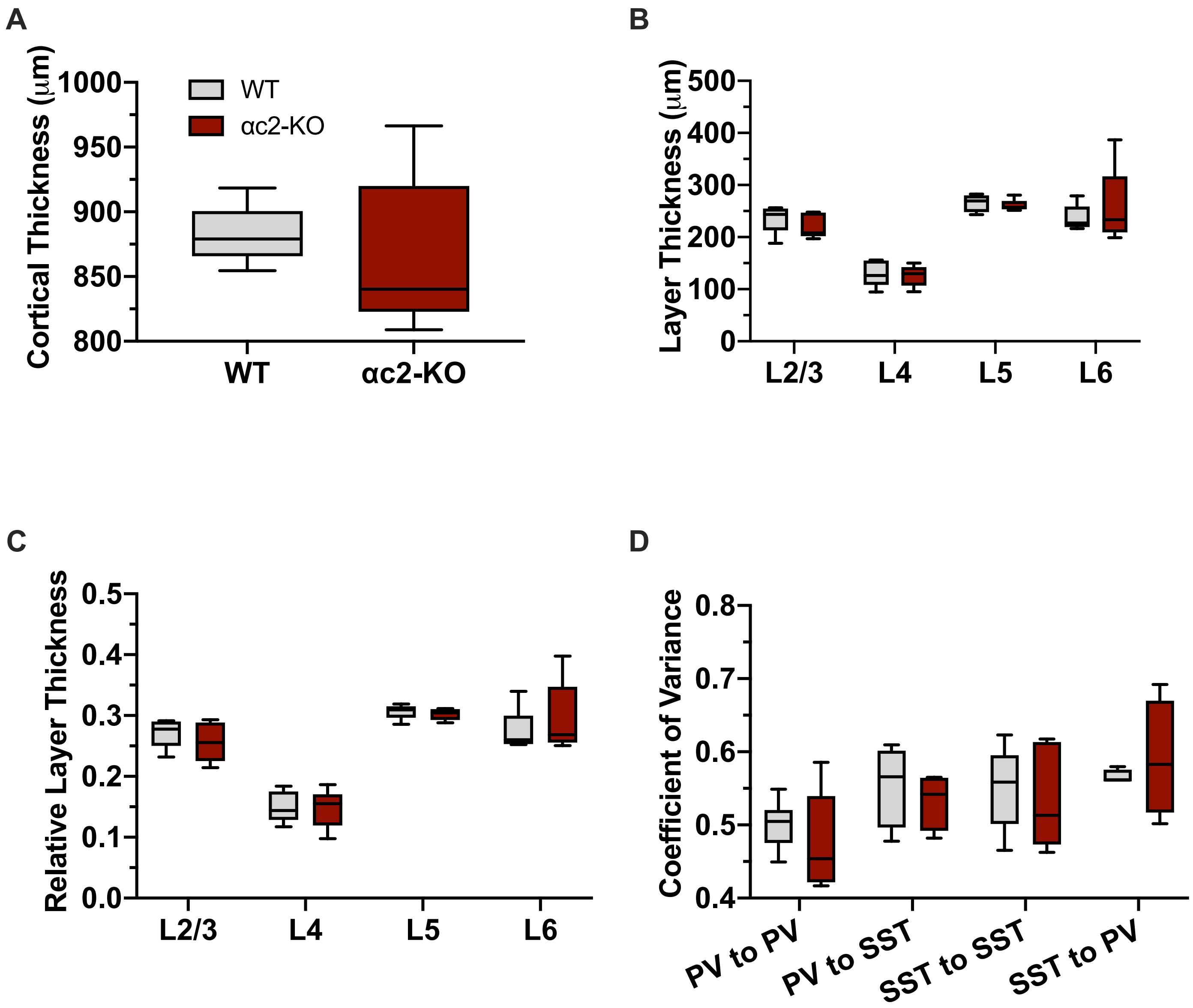

Supplemental Figure 9

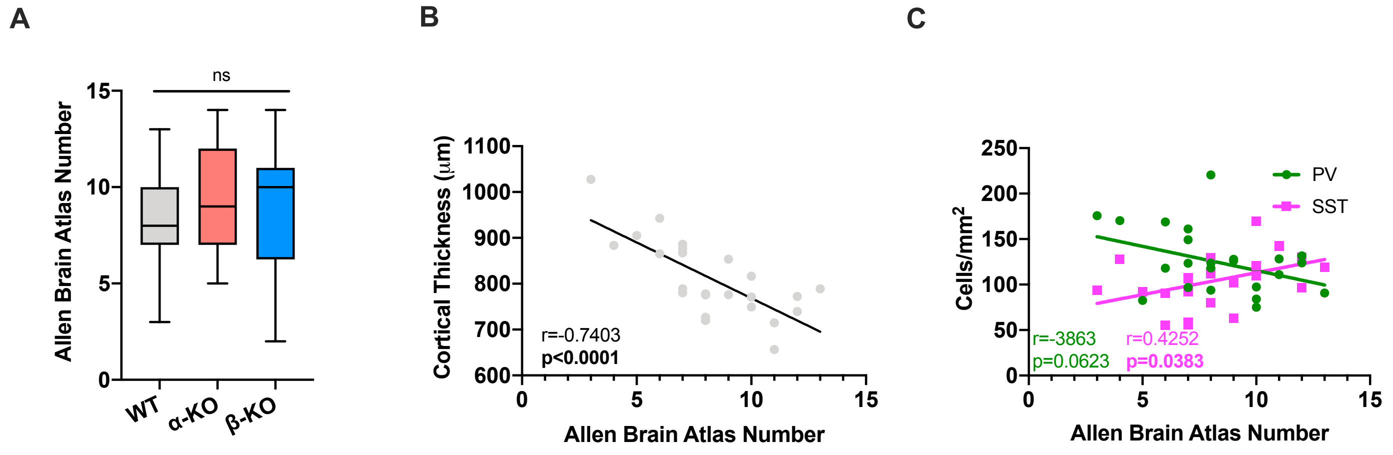

Supplemental Figure 10

Glossary

ABAN: Allen Brain Atlas Number
CV: Coefficient of variance of the nearest neighbor distance
DMCT: Dunnett's multiple comparisons test
L2/3, L4, L5, L6: Cortical layers 2/3, 4, 5, 6
LtR: Left to right
NND: Nearest neighbor distance
P3, P7, P13: Post-natal day 3, 7, 13
PCF: Paired correlation function
PV: Parvalbumin
S1: Primary somatosensory cortex
SMCT: Sidak's multiple comparisons test
SST: Somatostatin
V1: Primary visual cortex
WT: Wild Type
α-KO: Alpha clustered protocadherin knockout mouse
αc2-KO: Protocadherin αc2 knockout mouse
β-KO: Beta clustered protocadherin knockout mouse

Supplemental Figure Legends

Supplemental Figure 1-Semi-automated image quantification pipeline for spatial analysis of cINs
A-Representative fluorescent image of WT V1 labeled with DAPI (blue), anti-PV (green), and anti-SST (magenta) ; scale bar=200μm
B-Workflow for DAPI-containing channel. L: Representative fluorescent image of DAPI. R: The density of DAPI stained nuclei is used to manually draw layers onto the image. The XY coordinates of these layers are saved.
C-Workflow for PV-containing channel. LtR: Original image, image after difference of Gaussians, image after CLAHE, binarized image with outlines of size and circularity gated particles, final cell count after manual quality control overlaid on cell image
D-Workflow for SST-containing channel. LtR: Original image, image after difference of Gaussians, image after CLAHE, binarized image with outlines of size and circularity gated particles, final cell count after manual quality control overlaid on cell image
E-Final image generated in Spatstat using XY coordinates obtained from images

Supplemental Figure 2-Measurements from S1 are not significantly different from V1 except for cortical thickness
A-PV and SST cell density, WT V1 vs. S1. cIN density does not significantly differ between V1 (yellow) and S1 (orange) cortex (PV: $p=0.2605$, SST: $p=0.8004$, unpaired T-tests)
B-Relative proportion of PV cells in each layer, WT V1 vs. S1. The laminar distribution of PV cells was not different between V1 and S1 (LtR: $p=0.7889$, >0.999, >0.999, 0.7327, 2-way RM-ANOVA with SMCT)

C-Relative proportion of SST cells in each layer, WT V1 vs. S1. The laminar distribution of SST cells was not different between V1 and S1 (LtR: p=0.9939, p=0.2343, p=0.9416, p=0.3985, 2-way RM-ANOVA with SMCT)
D-CV between PV-PV, PV-SST, SST-SST, SST-PV pairs, WT V1 vs. S1. The CV was not different between V1 and S1 (LtR: p=0.2394, p=0.8499, p=0.9993, p=0.2153, 2-way RM-ANOVA with SMCT)
E-Density of PV cells in each layer, WT V1 vs. S1. PV density was not different in any layer between V1 and S1 (LtR: p=0.6495, p=0.9957, p=0.3367, p=0.4709, 2-way RM-ANOVA with SMCT)
F-Density of SST cells in each layer, WT V1 vs. S1. SST density was not different in any layer between V1 and S1 (LtR: p=0.9689, p=0.1389, p=0.9998, p=0.9991, 2-way RM-ANOVA with SMCT)
G-Cortical thickness, WT V1 vs. S1. Each cortical layer was significantly thicker in S1 compared to V1 (LtR: p=0.0002, p=0.0104, p=0.0019, p=0.0045, 2-way RM-ANOVA with SMCT)
H-Relative cortical thickness, WT V1 vs. S1. Proportionally, cortical layers were similar in V1 and S1 (LtR: p=0.2508, p=0.9760, p=0.9989, p=0.0978, 2-way RM-ANOVA with SMCT)
I-Fraction of all cells that were PV or SST, WT V1 vs. S1. The fraction of all cells that were PV or SST was not different between V1 and S1 (PV: p=0.4455, SST: p=0.4463, 2-way ANOVA with SMCT)

Supplemental Figure 3-Nearest neighbor distance is significantly negatively correlated with cell density
A-Correlation between WT PV-PV NND and PV density, V1. WT PV NND and PV density are significantly negatively correlated (r=-0.8533, p=0.0146, simple linear regression)
B-Correlation between WT SST-SST NND and SST density, V1. WT SST NND and SST density are significantly negatively correlated (r=-0.9870, p<0.0001, simple linear regression)
C-Correlation between α-KO PV-PV NND and PV density, V1. α-KO PV NND and PV density are significantly negatively correlated (r=-0.8690, p=0.0011, simple linear regression)
D-Correlation between α-KO SST-SST NND and SST density, V1. α-KO SST NND and SST density are significantly negatively correlated (r=-0.9534, p<0.0001, simple linear regression)
E-Correlation between β-KO PV-PV NND and PV density, V1. β-KO PV NND and PV density are significantly negatively correlated (r=-0.9504, p=0.0003, simple linear regression)
F-Correlation between β-KO SST-SST NND and SST density, V1. β-KO SST NND and SST density are significantly negatively correlated (r=-0.9393, p=0.0005, simple linear regression)

Supplemental Figure 4-Loss of cPcdh diversity alters laminar distribution in S1
A-Density of PV and SST cells in S1, WT vs. α-KO v β-KO. Cell density is not significantly different between genotypes in S1 (PV: WT v α-KO p=0.5756, WT v β-KO p=0.9529; SST: WT v α-KO p=0.8033, WT v β-KO p=0.8775, 1-way ANOVAs with DMCT)
B-NND between PV-PV, PV-SST, SST-SST, SST-PV pairs in S1, WT vs. α-KO vs. β-KO. Nearest neighbor distance is not significantly different between genotypes in S1 (PV-PV: WT v α-KO p=0.4432, WT v β-KO p=0.7229; PV-SST: WT v α-KO p=0.4325, WT v β-KO p =0.6701; SST-SST: WT v α-KO p=0.5401, WT v β-KO p=0.6184; SST-PV: WT v α-KO p=0.5659, WT v β-KO p=0.9355, 2-way RM-ANOVA w SMCT)

C-CV between PV-PV, PV-SST, SST-SST, SST-PV pairs in S1, WT vs. α-KO vs. β-KO. CV is not significantly different between genotypes in S1 (PV-PV: WT v α-KO p=0.8089, WT v β-KO p=0.8547; PV-SST: WT v α-KO p=0.6229, WT v β-KO p =0.2419; SST-SST: WT v α-KO p=0.9458, WT v β-KO p=0.5447; SST-PV: WT v α-KO p=0.6620, WT v β-KO p=0.2522, 2-way RM-ANOVA w SMCT)
D-Relative proportion of PV cells in each cortical layer in S1, WT vs. α-KO vs. β-KO. The relative amount of PV cells was increased in L6 in the α-KO. (L2/3: WT v α-KO p=0.7939, WT v β-KO p=0.9751; L4: WT v α-KO p=0.9996, WT v β-KO p =0.9767; L5: WT v α-KO p=0.6133, WT v β-KO p=0.2923; L6: WT v α-KO p=0.0394, WT v β-KO p=0.3091, 2-way RM-ANOVA w SMCT)
E- Relative proportion of SST cells in each cortical layer in S1, WT vs. α-KO vs. β-KO. The relative amount of SST cells was decreased in L5 of the β-KO. SST trended up in L2/3 in α-KO and β-KO, but these p values were barely above the statistical significance threshold. Similarly, L5 of α-KO trended lower. (L2/3: WT v α-KO p=0.1129, WT v β-KO p=0.0739; L4: WT v α-KO p=0.9290, WT v β-KO p=0.7582; L5: WT v α-KO p=0.1013, WT v β-KO p=0.0435; L6: WT v α-KO p=0.3883, WT v β-KO p=0.2658, 2-way RM-ANOVA w SMCT)
F-Density of PV cells in each cortical layer in S1, WT vs. α-KO vs. β-KO. PV density was not different across genotypes (L2/3: WT v α-KO p=0.3278, WT v β-KO p=0.9807; L4: WT v α-KO p=0.7350, WT v β-KO p=0.9973; L5: WT v α-KO p=0.2343, WT v β-KO p=0.7368; L6: WT v α-KO p=0.9949, WT v β-KO p=0.9930, 2-way RM-ANOVA w SMCT)
G-Density of SST cells in each cortical layer in S1, WT vs. α-KO vs. β-KO. SST density was not different across genotypes (L2/3: WT v α-KO p=0.8482, WT v β-KO p=0.733; L4: WT v α-KO p=0.8541, WT v β-KO p=0.7157; L5: WT v α-KO p=0.3649, WT v β-KO p=0.5513; L6: WT v α-KO p=0.9982, WT v β-KO p=0.6889, 2-way RM-ANOVA w SMCT)

Supplemental Figure 5-Regularity of spacing is not altered by loss of cPcdh diversity
A-CV between PV-PV, PV-SST, SST-SST, SST-PV pairs in V1, WT vs. α-KO. CV is not significantly different between WT and α-KO (LtR: p=0.3317, p=0.1717, p=0.4059, p>0.9999, 2-way RM-ANOVA with SMCT)
B-CV between PV-PV, PV-SST, SST-SST, SST-PV pairs in V1, WT vs. β-KO. CV is not significantly different between WT and β-KO (LtR: p=0.8832, p=0.2590, p=0.9781, 0.5915, 2-way RM-ANOVA with SMCT)
C-PCF of PV-PV pairs in V1, WT vs. α-KO vs. β-KO (No p<0.05 for any distance, 2-way RM-ANOVA with SMCT)
D-PCF of SST-SST pairs in V1, WT vs. α-KO vs. β-KO (No p<0.05 for any distance, 2-way RM-ANOVA with SMCT)

Supplemental Figure 6-The α-KO is thicker in both V1 and S1 compared to WT
A-Thickness of V1, WT v α-KO vs. β-KO. The α-KO cortex is thicker than WT cortex in V1 (WT v α-KO p=0.0066, WT v β-KO p=0.1711, 1-way ANOVA with DMCT)
B-Laminar thickness of V1, WT vs. α-KO vs. β-KO.In V1, L2/3 and L6 of the α-KO cortex is thicker than WT (L2/3: WT v α-KO p=0.0001, WT v β-KO p=0.0662; L4: WT v α-KO p=0.2799, WT v β-KO p=0.7861; L5: WT v α-KO p=0.0835, WT v β-KO p=0.5253; L6: WT v α-KO p=0.0358, WT v β-KO p=0.7198, 2-way RM-ANOVA w SMCT)

C-Relative laminar thickness of V1, WT v α-KO v β-KO. L2/3 in the α-KO is increased relative to other layers but other layers are proportionally unaltered (L2/3: WT v α-KO $p=0.0176$, WT v β-KO $p=0.3797$; L4: WT v α-KO $p>0.9999$, WT v β-KO $p=0.9985$; L5: WT v α-KO $p=0.3097$, WT v β-KO $p=0.8554$; L6: WT v α-KO $p=0.8392$, WT v β-KO $p=0.7870$, 2-way RM-ANOVA w SMCT)
D-Thickness of S1, WT v α-KO vs. β-KO. The α-KO cortex is thicker than WT cortex in S1 (WT v α-KO $p=0.0163$, WT v β-KO $p=0.2243$, 1-way ANOVA with DMCT)
E-Laminar thickness of S1, WT vs. α-KO vs. β-KO. In S1, L2/3 is thicker in both the α-KO and β-KO. L5 of the α-KO was thicker than β-KO but not statistically significant compared to WT. (L2/3: WT v α-KO $p=0.0086$, WT v β-KO $p=0.0194$; L4: WT v α-KO $p=0.062$, WT v β-KO $p=0.1816$; L5: WT v α-KO $p=0.2246$, WT v β-KO $p=0.9741$; L6: WT v α-KO $p=0.098$, WT v β-KO $p=0.3574$, 2-way RM-ANOVA w SMCT)
F-Relative laminar thickness of V1, WT vs. α-KO vs. β-KO. Proportionally, most layers are unchanged compared to WT, except L5 in the β-KO which is reduced (L2/3: WT v α-KO $p>0.9999$, WT v β-KO $p=0.5854$; L4: WT v α-KO $p=0.6454$, WT v β-KO $p>0.9999$; L5: WT v α-KO $p=0.9136$, WT v β-KO $p=0.0423$; L6: WT v α-KO $p=0.494$, WT v β-KO $p=0.7135$, 2-way RM-ANOVA w SMCT)

Supplemental Figure 7-Laminar distribution is not altered at P3 or P7, mutant phenotype emerges at P13

A-Thickness of V1 at P3, P7, and P13, Nkx2.1^{Cre}; Ai9; cPcdh^{WT} vs. α-KO. Cortical thickness increases over time but is not different between WT and α-KO (WT v α-KO: P3 $p=0.7857$, P7 $p=0.9623$, P13 $p=0.9341$; WT P3 v P7: $p<0.0001$ WT P7 v P13: $p=0.0101$, 2-way RM-ANOVA with SMCT)
B- CV between tdTomato+ cells in V1 at P3, P7, and P13, Nkx2.1^{Cre}; Ai9; cPcdh^{WT} vs. α-KO. CV is not different between WT and α-KO at any timepoint (LtR: $p=0.9962$, $p=0.8579$, 2-way RM-ANOVA with SMCT)
C-Relative proportion of tdTomato+ cells in each layer of V1 at P3, Nkx2.1^{Cre}; Ai9; cPcdh^{WT} vs. α-KO. The relative distribution of tdTomato+ cells is not different at P3 (LtR: $p=0.2558$, $p=0.1327$, $p=0.7794$, 2-way RM-ANOVA with SMCT)
D-Relative proportion of tdTomato+ cells in each layer of V1 at P7, Nkx2.1^{Cre}; Ai9; cPcdh^{WT} vs. α-KO. The relative distribution of tdTomato+ cells is not different at P7 (LtR: $p=0.9754$, $p=0.8558$, $p=0.9885$, 2-way RM-ANOVA with SMCT)
E-Relative proportion of tdTomato+ cells in each layer of Nkx2.1^{Cre}; Ai9; cPcdh^{WT} V1 at P3, P7, P13. In the WT, the relative amount of tdTomato+ cells in L2/3 decreases over time (L2-4: P3 v P13 $p=0.0276$, P3 v P7 $p=0.8806$, P7 v P13 $p =0.0528$; L5: P3 v P13 $p=0.2079$, P3 v P7 $p=0.8423$, P7 v P13 $p=0.3415$; L6: P3 v P13 $p=0.1594$, P3 v P7 $p>0.9999$, P7 v P13 $p=0.1084$, 2-way RM-ANOVA with SMCT)
F- Relative proportion of tdTomato+ cells in each layer of Nkx2.1^{Cre}; Ai9; α-KO V1 at P3, P7, P13. In the α-KO, the relative amount of tdTomato+ cells in L5 decreases over time (L2-4: P3 v P13 $p=0.1399$, P3 v P7 $p=0.5859$, P7 v P13 $p =0.2741$; L5: P3 v P13 $p=0.0250$, P3 v P7 $p=0.4807$, P7 v P13 $p=0.2879$; L6: P3 v P13 $p=0.9781$, P3 v P7 $p=0.9714$, P7 v P13 $p=0.9298$, 2-way RM-ANOVA with SMCT)

Supplemental Figure 8-Pcdh-αc2 is expressed at relatively high levels in PV and SST subtypes

A-cPcdh RNA expression in PV cells dissected from adult V1
B-cPcdh RNA expression in SST cells dissected from adult V1
C-Pcdh-αc2 expression in PV subtypes defined by Tasic 2018
D-Pcdh-αc2 expression in SST subtypes defined by Tasic 2018

Supplemental Figure 9-Cortical thickness and CV are unaltered in the αc2-KO

A-Thickness of P30 V1, WT vs. αc2-KO. Cortical thickness is not significantly altered in the αc2-KO (p=0.5832, unpaired T-test)
B-Laminar thickness of P30 V1, WT vs. αc2-KO. Laminar thickness is not significantly altered in the αc2-KO (LtR: p=0.8684, 0.9958, 0.9811, 0.9711, 2-way RM-ANOVA with SMCT)
C-Relative laminar thickness of P30 V1, WT vs. αc2-KO. Proportionally, layers are not significantly altered in the αc2-KO (LtR: p=0.8948, p=0.9997, p=0.9598, p=0.9463, 2-way RM-ANOVA with SMCT)
D-CV between PV-PV, PV-SST, SST-SST, SST-PV pairs in P30 V1, WT vs. αc2-KO. CV is not different for any cell pair examined (LtR: p=0.9075, p=0.9587, p=0.9787, p=0.9702, 2-way RM-ANOVA with SMCT)

Supplemental Figure 10-Comparison of Allen Brain Atlas Numbers of images within dataset

A-Comparison of images making up the WT, α-KO, and β-KO datasets. These did not significantly differ in their representation of certain Allen Brain Atlas numbers (ABAN) (LtR: p=0.3927, p=0.6765, p=0.9636, 1-way ANOVA with SMCT)
B-Correlation between WT cortical thickness and ABAN. Cortical thickness negatively correlates with ABAN (r=-0.7403, p<0.001, simple linear regression)
C-Correlation between WT PV and SST densities and ABAN. PV density is not significantly correlated with ABAN, while SST density is modestly positively correlated with ABAN (PV: r=-0.3863, p=0.0623; SST: r=0.4252, p=0.0383, simple linear regression)
